# Role of CTLA4 and pSTAT3 Immunostaining in Prognosis and Treatment of the Colorectal Carcinoma 

**DOI:** 10.30699/IJP.2024.2009619.3158

**Published:** 2024-03-29

**Authors:** Dina Mohamed Allam, Hend Kasem, Amira Hegazy, Shereen F Mahmoud

**Affiliations:** 1 Department of Pathology, Faculty of Medicine, Menoufia University, Shebin El Kom, Egypt; 2 Department of Clinical Oncology and Nuclear Medicine, Faculty of Medicine, Menoufia University, Egypt

**Keywords:** Colorectal carcinoma, CTLA4, Immunotherapy, Prognosis, pSTAT3, Targeted therapy

## Abstract

**Background & Objective:**

Colorectal carcinoma (CRC) is the third leading cause of cancer-caused death worldwide and constitutes about 6.48% of all malignancies in Egypt. Studying the molecular profile of CRC is essential for developing targeted therapies. STAT3 and CTLA4 expression are considered as molecular abnormalities involved in the CRC progression and chemo-resistance. Therefore, they could be used as potential therapeutic targets. This study aimed to evaluate pSTAT3 and CTLA4 expression levels and their possible roles as prognostic and predictive biomarkers in CRC using immunohistochemistry (IHC).

**Methods:**

This retrospective study included 113 CRC patients. Tissue microarrays were constructed, followed by pSTAT3 and CTLA4 antibodies immunostaining. Their expression was assessed and compared with the clinicopathological parameters and survival data.

**Results:**

Both pSTAT3 and CTLA4 overexpression were significantly associated with poor prognostic parameters, such as the presence of distant metastasis (*P*=0.02 & 0.03), high grade (*P*<0.001 & 0.03), high mitotic count (*P*<0.001 & 0.03), high tumor budding group (*P*=0.008 & 0.04), infiltrating tumor border (*P*<0.001 & 0.007) respectively, and advanced pathological stage with pSTAT3 (*P*=0.02). A significant association was found between overexpression of both markers and short overall survival. Correlations between the H-score of pSTAT3 and CTLA4 in CRC showed a significant positive correlation (*P*<0.001).

**Conclusion:**

STAT3 and CTLA4 positivity may be linked to the development and progression of the CRC, and they may provide potential prognostic indicators and therapeutic targets for CRC patients.

## Introduction

One of the most prevalent malignant tumors of the digestive system is colorectal cancer (CRC). Worldwide, CRC is the second most frequent cancer in women and the third most common cancer in men. It is regarded as the third most common cause of cancer-related death (1).

In Egypt, there is a progressive increase in incidence and deaths, especially among patients younger than 50 years old. According to the National Cancer Institute Registry, CRC represents 6.48% of total malignancies between 2000 and 2011 (2). About 3055 CRC cases were anticipated in Egypt in 2015, and 4840 were anticipated in 2020 (3).

It is possible to divide CRC into various subtypes based on their distinct clinical, molecular, and morphological abnormalities (4,5). Patients show great variation in prognosis and response to therapies (6).

Most of the CRCs originate from cancer stem cells (CSC) within the colonic epithelium, accumulating progressive genetic and epigenetic changes. These changes lead to impaired gene expression and/or function, thereby favoring activation of oncogenes and downregulation of tumor suppressor genes (7,8).

CRC treatment strategies include endoscopic and surgical treatment, radiotherapy for rectal cancers, local therapies for metastatic disease, systemic chemotherapy, novel targeted agents, and immunotherapy (9). Immunotherapy and targeted therapy are currently considered the two most important therapeutic options in selecting effective patient criteria, offering some patients with specific molecular characteristics prolonged survival and a reduction in progression. (10)

JAK-STAT pathway is an important oncogenic signaling cascade that includes the family of Janus-Kinase (JAK) non-receptor tyrosine kinase and the signal transducer of activation of transcription (STAT) (11).

Seven proteins make up the STAT family, but STAT3 is the one that has a significant role in the development of cancer. (12)

Numerous human malignancies, including breast cancer, prostatic cancer, multiple myeloma, and head and neck tumors, have been linked to STAT3 (13,14).

Signal transducers and activators of transcription 3 (STAT3) also have been most reported with CRC initiation and development (15). STAT3 controls the immune response against malignancies and represents a unique feature in CRC (16), as well as tumor growth, invasion, and migration (17). These characteristics represent STAT3's potential as a therapeutic target; nevertheless, for STAT3 inhibitors to enter clinical trials, the mechanisms behind these characteristics still need to be thoroughly understood (18).

Immune checkpoints (ICP) affect cancer cell immune evasion and are expressed by tumors to escape T-cell mediated lysis. Many ICP molecules are targeted for activating the immune system and unleashing anti-tumor immunity to eliminate malignant cells. The most recent ICP targets are cytotoxic T-lymphocyte antigen 4 (CTLA 4) (19, 20).

As a crucial component of the malignant process and a novel target for cancer therapy, the immune system's role as an anti-tumor has recently been focused on in studies. (21) The current study aimed to examine the links between pSTAT3 and CTLA-4 immunohistochemical markers and CRC.

This study aimed to evaluate pSTAT3 and CTLA4 expression and their possible roles as prognostic and predictive biomarkers in colorectal carcinoma using immunohistochemistry (IHC).

##  Material and Methods

This retrospective study included 113 CRC cases. Formalin-fixed, paraffin-embedded (FFPE) tissue blocks were retrieved from the archival material obtained from the Pathology department, Faculty of Medicine, Menoufia University, spanning the period from 2015 to 2022.

This study was performed after approval (10/2022PATH32) by the Ethical Committee of the Faculty of Medicine, Menoufia University, and the study was conducted by the Declaration of Helsinki in 1975 and modified in 2000.

Clinical data of the CRC cases included gender, age, family history, clinical presentation, initial presentation of intestinal obstruction, tumor site, tumor markers (CEA, CA19.9), genetic testing (KRAS, MSI), number of lines of chemotherapy, type of chemotherapy, treatment received, time of surgery, the onset of metastatic disease, number of metastatic lesions, local treatment of oligometastatic disease (OMD), response and survival data were collected from patients' files at Oncology Department, Faculty of Medicine, Menoufia University, health insurance, and private clinics.

Response to treatment was assessed using RECIST version 1.1. (22)

Overall Survival (OS) time was calculated in months from the date of diagnosis and ended with the patient's death or the date of the last follow-up visit (23).

Progression-free survival (PFS) time was calculated in months from the date of diagnosis to the date of tumor progression. Progression was defined as relapse of the tumor in the operative field, regional lymph nodes, and/or distant metastasis (23).

PFS is a clinical endpoint for treatments to manage more advanced metastatic malignancies (24).

**Histopathological Evaluation**

Cases were selected based on the availability of paraffin blocks obtained for recutting. All selected cases were surgically colectomy specimens. The hematoxylin and eosin (H&E) stained slides were examined for confirmation of the diagnosis and evaluation of histopathological findings, including tumor location, tumor size, gross morphology, gross perforation, histopathological type, and tumor grade (low and high grade) according to the WHO classification of GIT tumors 5^th^ edition (25), lymphovascular invasion, perineural invasion, margins, mitotic, apoptotic count**, **tumor budding, and tumor infiltrating-lymphocytes (26).

Staging of the tumor was performed according to the TNM American Joint Committee on Cancer-Union International Center AJCC 8th edition (27).

Age, size of tumor, and mitotic count were divided into two groups using their median.

Tissue microarrays (TMA) were prepared from the collected paraffin blocks by labeling the selected viable tumor foci in H&E-stained slides, and the corresponding block was marked at the same selected foci. Three tissue cores with a diameter of 2 mm were punched from the donor block using a manual tissue array needle (Beecher Instruments, Silver Spring, USA). The retrieved tissue cores were arrayed onto a recipient block. A map is created to show the origin and location of each core (28).

Two sections, each 4 µm slices, were cut from constructed 4 TMA blocks and then immunohistochemically stained with pSTAT3 and CTLA4 antibodies. 

**Immunohistochemical Staining**

Immunostaining was conducted using the streptavidin-biotin amplification system with diaminobenzidine (DAB) as the suitable substrate/chromogen reagent. The slides were then dewaxed and rehydrated. Antigen retrieval was achieved by boiling citrate buffer saline (pH 6) and cooling to an average temperature. The primary antibody was incubated overnight at room temperature, after which the secondary antibody (Envision, FLEX, code 8002, Dako) was added. DAB was employed as a chromogenic substrate, and Mayer's hematoxylin was used as a counterstain. The two primary antibodies used were against Phospho-STAT3 antibody (A rabbit polyclonal antibody raised against amino acid residues around human pSTAT3 (phosphor Tyr705) GTX118000, GeneTex, USA, dilution 1:50) and CTLA4 (A rabbit polyclonal anti-human antibody (Chongqing Biospes), Catalog # YPA1004, Concentrated form (50 micron) with a dilution of 1:100).

Renal cell carcinoma was used as a positive control for the pSTAT3 antibody, and normal human tonsil was used as a positive control for the CTLA4 antibody.

A slide without the primary antibody was included in each run as a negative control.

**Immunohistochemical Assessment**

The immunohistochemical expression of pSTAT3 and CTLA4 was evaluated by two pathologists. Their expression was assessed in the malignant and stromal cells regarding the staining state (positive or negative) and subcellular localization in malignant cells. Positive pSTAT3 expression cells showed nuclear brownish coloration (29)**. **Cells with CTLA4 expressions showed brownish membrano-cytoplasmic staining (30). Cases were considered positive if any malignant or stromal cells showed definite staining.

The expression was semi-quantitatively scored using the H-score. The intensity of positive staining was scored by "H-score" as follows: 0 (no staining); 1 (slightly brown); 2 (moderately brown); and 3 (dark brown). The percentage of positively stained cells was determined. The H-score was calculated by multiplying both numbers. The final score has a numerical value range from 1 to 300 (31). Then, the cases were categorized into two groups using the median of 190.

**Statistical Analysis**

Data were collected, tabulated, and statistically analyzed using SPSS version 22 (SPSS Inc., Chicago, Ill., USA). Fisher's exact test (F), Qi square test (χ^2^), and Spearman correlation test were used. Survival analysis using a Log-rank test was also done. A P-value<0.05 is considered statistically significant, and a P-value<0.001 is considered highly significant.

## Results

Clinicopathological data of the colorectal carcinoma cases and demographic, clinical, and pathologic data of the investigated CRC cases are demonstrated in Table 1.

**Fig. 1:**
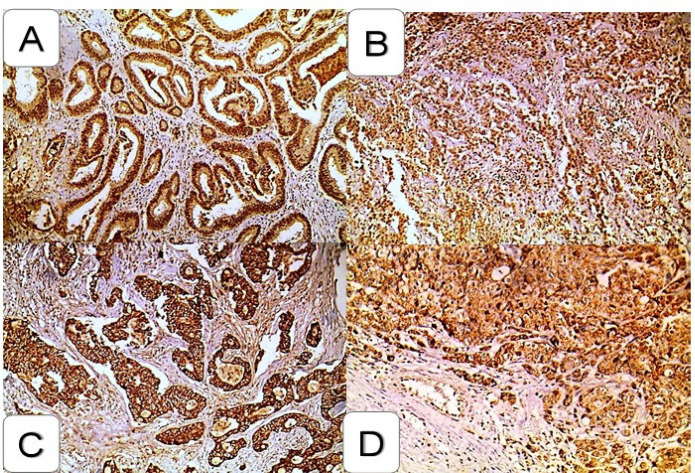
Immunohistochemical expression of pSTAT and CTLA4 in the colorectal carcinoma

Immunohistochemical staining results:

All the studied CRC cases showed pSTAT3 & CTLA4 expression; regarding pSTAT3 immunohistochemical staining results, it showed nuclear expression, while CTLA4 showed membrano-cytoplasmic expression (Figure 1).

**The Relationship between H-score of pSTAT3 Expression and Clinicopathological Data**

pSTAT3 overexpression showed a statistically significant association with old age (*P*=0.02), lymph node metastasis (*P*=0.03), distant metastasis (*P*=0.02), advanced AJCC tumor stage (*P*=0.02), and high-grade tumors (*P*<0.001) with regards to the relationship between pSTAT3 H-score and the clinicopathological parameters of prognostic significance.

Moreover, there was a statistically significant association between higher H score and positive tumor margins (*P*=0.02), high mitotic count (*P*=0.001), high tumor budding score (P=0.008), and infiltrating tumor borders (*P*<0.001) (Table 2).

**The Relationship Between H-score of CTLA4 Expression and Clinicopathological Data**

CTLA4 overexpression showed a statistically significant association with old age (*P*=0.03), T stage (*P*=0.02), distant metastasis (*P*=0.03), and high grade (*P*=0.03) as regards the relationship between CTLA4 H-score and the clinicopathological parameters of prognostic significance.

Moreover, there was a statistically significant association between higher H score of CTLA4 and high mitotic count (*P*=0.03), high tumor budding score (*P*=0.04), infiltrating tumor borders (*P*=0.007), initial presentation with intestinal obstruction (*P*=0.04) and KRAS mutation (*P*=0.04) (Table 2). 

**Survival Analysis**

The survival data was available in all the CRC cases, with the last follow-up date in July 2022. The overall survival of the cases ranged from 7 to 44 months, with 20.4 months as a mean. Thirty-one (27.4%) patients were dead. The mean PFS was 17.44 months (Table 1).

**Overall Survival (OS)**

**Fig. 2:**
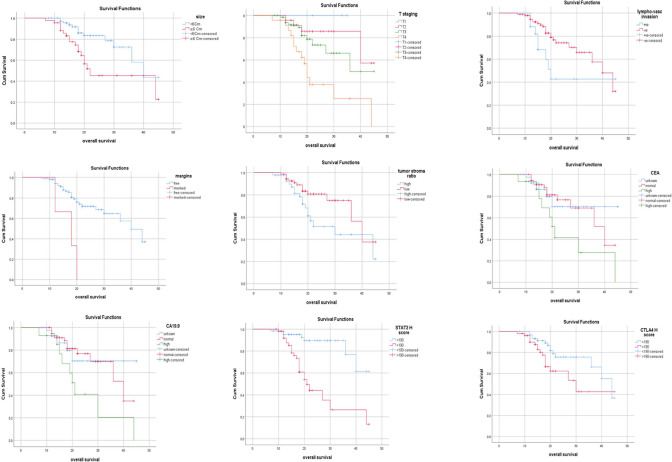
Kaplan-Meier overall survival curve in the malignant cases and significant parameters in univariate overall survival analysis

Univariate analysis of OS showed that there was a significant association between short OS and large size of tumor (*P*=0.006), deeper tumor invasion (*P*<0.001), positive lymphovascular invasion (*P*=0.04), free tumor margins (*P*<0.001), infiltrating tumor border (*P*=0.02), normal CEA and CA19.9 level (*P*=0.03, 0.02) respectively, high pSTAT3 immunostaining (*P*<0.001), and high CTLA4 immunostaining (*P*=0.03) (Figure 2). 

Multivariate analysis for OS showed that T3, T4 stage, Initial presentation of IO, and high STATA3 were independent prognostic factors of OS. Advanced T stage is the most independent prognostic factor (*P*<0.001) (Table 3).

**Progression Free Survival (PFS)**

**Fig. 3:**
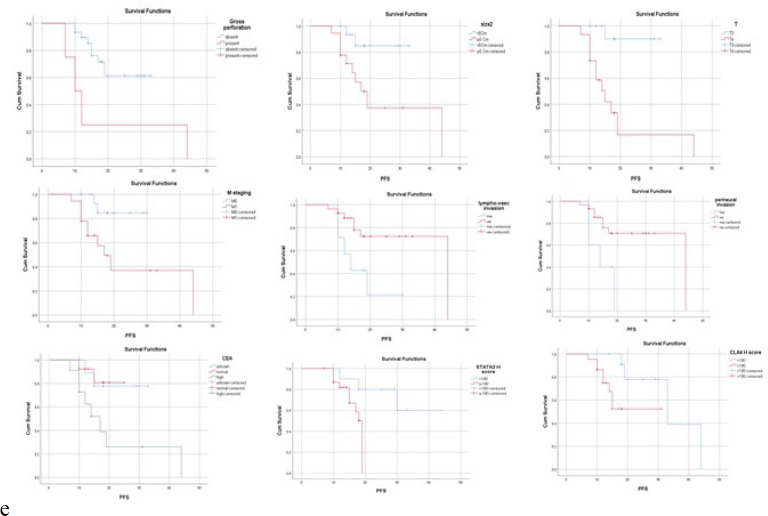
Kaplan-Meier Progression-free survival (PFS) curve in the malignant cases and significant parameters in univariate overall survival analysis

By univariate analysis of PFS, there was a significant association between PFS and gross perforation, size, tumor depth, metastasis stage, lymphovascular invasion, perineural invasion, margins, mitotic count, tumor stromal ratio, tumor border configuration, CEA, initial presentation of IO, STAT3, and CTLA4 score (Figure 3).

By multivariate analysis of PFS, it was found that the T4 stage, high mitotic count, and high STAT3 H-score were independent prognostic factors of PFS (Table 4).

There was a significant positive correlation between the H score of pSTAT3 and CTLA4 (*P*<0.001) (Table 5). 

**Table 1:** Clinicopathological data of the studied CRC patients (N=113)

Clinicopathological data	Number (%)
Gender	
Male Female	57(50.4) 56(49.6)
Age	
Mean± SD Range Median	52.04±14.36 21 - 86 52
Family History	
Present Absent	11(9.7) 102(90.3)
	
Clinical presentation	
Bleeding per rectum Abdominal pain Constipation Constipation+ Bleeding per rectum	44(38.9) 29(25.7) 39(34.5) 1(0.9)
Initial presentation of Intestinal obstruction	
Present Absent	30(26.5) 83(73.5)
Site of tumor	
RT. Colon LT. colon Rectum	48(42.5) 41(36.3) 24(21.2)
Mean± SD Range Median	6.22±3.55 1.8 – 20 6
Gross morphology	
Fungating mass Ulcer Infiltrating	48(42.1) 34(30.1) 31(27.4)
Gross perforation	
Present Absent	8(7.1) 105(92.9)
T Stage	
T1 T2 T3 T4	3(2.7) 23(20.4) 64(56.6) 23(20.4)
N Stage	
N0 N1 N2	60(53.1) 33(29.2) 20(17.7)
M Stage	
M0 M1	90(79.6) 23(20.4)
AJCC stage grouping	
I II III IV	21(18.6) 37(32.7) 32(28.3) 23(20.4)
Histopathological type	
Conventional adenocarcinoma Mucinous Mixed adenocarcinoma and mucinous carcinoma	81(71.1) 12(10.9) 20(17.7)
Grading	
High Low	29(25.7) 84(74.3)
Association with adenoma in bowel Present Absent	16(14.2) 97(85.8)
Lymphovascular invasion Positive Negative	17(15.0) 96(85.0)
Perineural invasion Positive Negative	12(10.6) 101(89.4)
Necrosis Present Absent	23(20.4) 90(79.6)
Margins Free Involved	110(97.3) 3(2.7)
Mitotic count Mean± SD Range Median	6.75±5.44 1 – 17 7
Tumor budding number Mean± SD Range	4.56±4.69 0 – 14
Tumor budding score High Intermediate Low	31(27.4) 22(19.5) 60(53.1)
Tumor border configuration Infiltrating Pushing	52(46.0) 61(54.0)
CEA Unknown Normal High	41(36.3) 56(49.6) 16(14.2)
CA19.9 Unknown Normal High	41(36.3) 58(51.3) 14(12.4)
KRAS Mutant Unknown	3(2.7) 110(97.3)
MSI Unknown Stable High	111(98.2) 1(0.9) 1(0.9)
No. of Chemotherapy lines 0 1 2	29(25.7) 54(47.8) 19(25.7)
Type of Chemotherapy Oxalobased Oxalo-based+folfiri Oxalo-based+xeloda	53(46.9 19(16.8) 10(8.8)
Treatment Palliative (surgery+CT+palliative RT) Surgery Surgery+CT Surgery+CT+avastin+maintenance xeloda,avastin+CRT Surgery+CT+CRT Surgery+CT+maintenance xeloda	1(0.9) 31(27.4) 59(62.2) 1(0.9) 19(16.8) 2(1.8)
Time of surgery After CT After CRT After CT+CRT Unfront	2(1.8) 17(15.0) 1(0.9) 93(82.3)
No. of metastasis 1 2 3 4 5 >5	3(2.7) 9(8.0) 3(2.7) 3(2.7) 2(1.8) 10(8.8)
Local treatment of OMD CRT RFA RT Surgery	1(0.9) 2(1.8) 1(0.9) 7(6.2)
Response CR PD PR DS	85(75.2) 20(17.7) 3(2.7) 5(4.4)
Synchronous or metachronous metastasis Synchronous Metachronous	23(76.7) 7(23.3)
Progression status Present Absent	20(17.7) (82.3)
PFS Mean± SD Range	17.44±8 7- 44
Survival status Alive Dead	82(72.8) 31(27.4)
Overall survival Mean± SD Range	20.44±8.41 7 – 44

**Table 2:** Relationship between the H score of STAT3 and CTLA4 H score and the clinicopathological parameters in the CRC cases (N=113)

Clinicopathological data	STAT3 H score		CTLA4 H score		
	Mean± SD	P-value	Mean± SD	P-value	
**Gender** Male Female	200±66.17 180.89±73	U=0.130	175.96±79.38 190.27±65.29	U=0.435	
**Age** <52 years ≥52 years	176.44±74.68 206.85±61.5	t =0.02*	154.2±68.6 182.6±70.8	U=0.033*	
**Clinical presentation** Bleeding per rectum Abdominal pain Constipation Constipation+Bleeding per rectum	200.2±72.1 166.7±72.8 199.4±63.5 160	F=0.172	163.98±63 159.5±80.7 175.3±70.6 175.26	F=0.298	
**Progression Status** Present Absent	208.8±60.8 187.2±71.6	U=0.218	174±86 166.5±67.5	U=0.589	
**Site of tumor** RT. colon LT. colon Rectum	189.5±71.4 188.8±70.5 197.7±69.1	F=0.870	169.3±74.1 173.8±68.6 154.6±68.8	F=0.567	
**Size** <6 ≥6	191.4±68.5 190.3±73.3	U=0.863	174.4±63.7 157.5±80.3	U=0.239	
**Gross morphology** Fungating mass Ulcer Infiltrating	179.7±65.9 196.6±67.1 202.3±78.7	KW=0.228	162.2±74.8 172.7±68.2 171.1±68.8	KW=0.786	
**Gross perforation** Absent Present	188.5±70.9 223.1±50.6	U=0.245	166.3±71.8 186.9±56.6	U=0.337	
**T Stage** T1 T2 T3 T4	206.7±57.7 166.74±68.9 191.33±74 212.2±55.9	KW=0.244	218.33±86.94 151.74±63.149 183.13±74.36 209.57±67.25	KW=0.024*	
**N Stage** N0 N1 N2	174.7±69.8 213±68 203.5±64.2	F=0.026*	163.3±69.3 179.2±71.9 162.5±75	KW=0.546	
**M Stage** M0 M1	183.2±71.3 221.3±56.7	U=0.016*	175.61±72.59 212.17±67.32	U=0.027*	
**AJCC staging** I II III IV	167±70.8 175.4±69.9 202.8±71 221.3±56.7	KW=0.016*	151.7±65.5 166.5±71.8 163.6±64 190.4±81	KW=0.216	
**Histopathological type** Conventional adenocarcinoma Mucinous Mixed	189.44±70.8 202.9±70.3 190±69.7	KW=0.430	169.2±72.5 174.6±73.7 158±64.3	KW=0.802	
**Grading** High Low	231.72±176.9 179.9±69.9	U<0.001**	190.3±81.1 160±65.6	U=0.03*	
**Association with adenoma in bowel** Present Absent	191.9±75.3 190.8±69.6	U=0.960	157.5±74.4 169.5±70.44	U=0.530	
**Lymphovascular invasion** Positive Negative	191.8±72.5 190.8±70	U=0.817	172.4±75.8 166.98±70.3	U=0.815	
**Perineural invasion** Positive Negative	205.4±66.8 189.3±70.6	U=0.505	183.8±71 165.9±70.9	0.403	
**Necrosis** Absent Present	190.4±71.2 193±66.8	U=0.954	169.6±66.7 160.9±86	U=0.622	
**Margins** Free Involved	189.1±70 258.3±23.6	U=0.019*	168.6±71 138.3±67.9	U=0.489	
**Mitotic count** <7 ≥7	157±65.8 183±77.3	U=0.001**	150.8±82.9 157.8±61.6	U=0.028*	
**Tumor budding score** High Intermediate Low	212.4±64.9 212.3±68.6 172.3±68.8	F=0.008*	188.4±76.4 158.6±63.5 160.5±69.3	U=0.038*	
**Tumor border configuration** Infiltrating Pushing	217.98±62.98 167.95±67.97	U<0.001**	201.63±73.36 167.21±68.97	U=0.007*	
**CEA** Unknown Normal High	184.2±66.9 188.3±73.8 217.8±61.9	KW=0.366	167.7±66.9 166.4±69.5 172.8±87.9		KW=0.873
**CA19.9** Unknown Normal High	184.2±66.8 194.4±73.8 196.8±66.6	KW=0.748	167.7±66.9 1655±67.9 177.5±95		KW=0.078
**Initial presentation of Intestinal obstruction** Yes No	208.5±64 184.6±71.4	U=0.101	188.3±78.5 160.4±66.7		U=0.037*
**KRAS** Mutant Unknown	245±31.2 189.5±70.3	U=0.234	200±17.3 166.9±71.5		t=0.049*

t = student t test U= Mann-Whitney test KW=Kruskal Wallis test F= One Way ANOVA

*P-value of < 0.05: statistically significant **P-value of < 0.001: statistically highly significant.

**Table 3:** The multivariate COX regression for detection of the independent factors affecting patient overall survival

Predictors (Independent variables)	Hazard ratio	95% CI (lower-upper)	P-value
**Size ≥ 6 cm**	2.070	0.754 – 5.683	0.158
**T Stage** **T2** **T3** **T4**	1.723 4.930 2.493	0.558 – 5.321 1.615 – 15.048 1.476 – 4.211	0.344 0.005* 0.001*
**Lymphovascular invasion Positive**	2.640	0.859 – 8.119	0.09
**Margins Involved**	3.229	0.726 – 14.370	0.124
**Tumor border configuration Infiltrating**	1.285	0.310 – 5.323	0.730
**High CEA**	1.541	0.423 – 5.616	0.512
**High CA19.9**	0.411	0.066 – 2.558	0.341
**Initial presentation of IO**	3.331	1.223 – 9.07	0.019*
**STAT3 H score (≥190)**	3.547	1.023 – 12.302	0.046*
**CTLA4 H score (≥190)**	1.460	0.546 – 3.904	0.451

**Table 4:** The multivariate COX regression for detection of the independent factors affecting patient PFS

Predictors (Independent variables)	Hazard ratio	95% CI (lower-upper)	P-value
**Gross perforation**	0.565	0.005 – 5.785	0.630
**Size** **≥6 cm**	1.964	0.342 – 11.271	0.449
**T Stage** **T4**	11.483	1.012 – 130.319	0.04*
**M Stage** **M1**	1.132	0.161- 7.934	0.901
**Positive Lymph vascular invasion**	1.109	0.262 – 4.695	0.888
**Positive perineural invasion**	1.779	0.412 – 7.689	0.440
**Involved Margins**	4.981	0.287-86.445	0.270
**Mitotic count** **≥7**	22.109	2.104-232.286	0.01*
** Infiltrating Tumorborder configuration**	0.027	0- 5.88	0.189
**High CEA**	1.162	0.157 – 8.593	0.883
**Initial presentation of IO**	0.350	0.07 – 1.754	0.202
**STAT3 H score** **≥190**	21.762	1.219 – 388.458	0.036*
**CTLA4 H score** **≥190**	2.669	0.424 – 16.785	0.296

PFS: progression-free survival

CI: Confidence interval

T stage: tumor depth stage

M stage: presence of the tumor metastasis

IO: intestinal obstruction

*P-value<0.05: statistically significant

**P-value<0.001: statistically highly significant.

**Table 5:** Correlation between STAT3 H score and CTLA4 H score

Variables	STAT3 H score			CTLA4 H score	
	rs	P-value		rs	P-value
**STAT3 H score **	--		--	0.557	<0.001**
**CTLA4 H score**	0.557		<0.001**	---	---

rs: spearman correlation test *P value<0.05: statistically significant **P value<0.001: statistically highly significant.

- Strong nuclear expression of pSTAT in the low-grade colorectal carcinoma. (IHCX200)
- Strong nuclear expression of pSTAT in high-grade colorectal carcinoma. (IHCX200)
- Strong membrano-cytoplasmic expression of CTLA4 in low-grade colorectal carcinoma. (IHCX200)
- Strong membrano-cytoplasmic expression of CTLA4 in the high-grade colorectal carcinoma. (IHCX200)

## Discussion

CRC is one of the most prevalent malignant tumors of the digestive system and the third leading cause of cancer-caused death (1). Highlight the importance of developing novel and efficient therapeutic regimens for CRC (32).

Further, the current treatment options with chemotherapy have provided moderate results in terms of efficacy and remission, warranting novel therapeutic options. Recently, immunotherapy has provided significant hope in cancer therapy (21).

In over 70% of human malignancies, STAT3, a transcriptional modulator of oncogenic signaling, is constitutively active. STAT3 activation in CRC is linked to poor clinical outcomes, suggesting a possible function as a therapeutic target (33). Since no inhibitors have been authorized for treating CRC or other cancers, the development of pSTAT3 inhibitors is an important area of research (18, 34).

In the current study, pSTAT3 showed positive nuclear expression in all the CRC cases with high H-score expression in more than half of the cases, in agreement with Gargalionis *et al.*, who also found positive nuclear pSTAT3 expression in all the CRC cases and reported high expression in half of the cases (35).

Numerous investigations were conducted to evaluate the significance of pSTAT3 overexpression in prognosis of numerous malignancies. Most of these studies revealed that pSTAT3 overexpression in many patients has a poor prognostic value. (36, 37) Other studies found that pSTAT3 overexpression is related to better prognosis and favorable outcomes, particularly in thyroid and breast carcinoma (38, 39).

Our study revealed that a high pSTAT3 H-score is significantly associated with poor prognostic factors, such as advanced AJCC staging, tumor budding, and short overall survival. These findings were in agreement with the systematic review and meta-analysis by Zhang *et al.* (2016), where high pSTAT3 expression level is associated with diverse clinicopathologic features, including lymph node metastasis (40). Also, another study showed that pSTAT3 expression is significantly associated with higher mortality (41).

Kusaba *et al.* also found a positive correlation between STAT3 expression and decreased overall survival, TNM stage, and depth of tumor invasion consistent with our result (42), as did another meta-analysis study conducted on patients with digestive system malignancies (43).

pSTAT3 role in inhibiting tumor cell apoptosis, promoting tumor invasion and metastasis, and immune evasion can be attributed to the positive correlation between pSTAT3 over expression advanced pathological tumor stage and grade. (37) It was proved that to evaluate the real activity of the pSTAT3 protein, it is necessary to quantify its activity in the nucleus, where it has a crucial role in carcinogenesis and progression of the disease rather than its overall activity (44).

STAT3 is activated in the CRC, among other types of malignancy, and is also found as a regulator of CRC cell resistance to chemo-radiotherapy (45). Our findings demonstrate that pSTAT3 expression is positively linked to greater resistance, reflected in a poor prognosis and short PFS.

The PD-1/PD-L1 and CTLA-4 pathways are currently recognized as representing essential players of immunotherapy for malignancies among various immune checkpoints, and "cancer immunotherapy" was chosen as Science's selection of Breakthrough of the Year 2013. (46) Immune checkpoint expression on the tumoral membrane aids the tumor's ability to evade host immune detection (47).

The surface of the regulator and effector T lymphocytes often expresses CTLA-4. Contrarily, Contardi *et al.* found that tumor cells also expressed CTLA-4 (48). Many researchers have also previously confirmed that CTLA-4 is widely expressed immunohistochemically in the tumor cells, and overexpression of CTLA-4 in the tumor cells has been linked to a worse prognosis in malignancies (47), consistent with our results. 

The present study revealed that CTLA4 showed membrano-cytoplasmic expression in the CRC and showed that cases with high H-score expression were significantly associated with poor prognostic factors, including the presence of distant metastasis, high grade, high mitotic count, tumor budding, presentation, and KRAS mutation and short survival. These findings are in agreement with Narayanan *et al.*, who described up-regulation of the CTLA4 intensity in the CRC tissues and its prediction role in tumor infiltration and bad prognosis (21). Hu *et al.*'s meta-analysis reported similar findings, which found a strong link between CTLA-4 in a Single Nucleotide Polymorphism (SNP) subset and overall survival (49).

Additionally, these findings concur with other CTLA4 studies in various malignant tumors, such as glioma tumors (50) and non-small cell lung cancer (51). This inhibitory signaling increased T cell activation's down-regulation, accelerating tumor progression and growth. Furthermore, other multiple studies demonstrated that persistent expression of CTLA 4 in the tumors correlated with tumor progression (52-54).

This could be explained by the fact that CTLA4 inhibits cytokine synthesis and T cell-mediated cytotoxicity to exert its immunosuppressive effects (55).

These results were opposite to that of those who demonstrated that high expression of CTLA4 was unexpectedly related to low grade, and this could be explained as CTLA 4 found on the tumor cells is functional in that it can specifically trigger an apoptotic effect after interaction with CD 80 and CD 86 ligands via caspases leading to decrease tumor growth (48) also Zhang *et al.* (2019) found that the tumor cell-intrinsic expression of CTLA 4 has a different function than that of checkpoint protein in T cells (51).

Recently, soluble intact anti-CTLA-4 antibody, which can enhance anti-tumor immunity by greatly boosting T-cell responses to both antigen and superantigen, has become a hotspot. The anti-tumor effects had been wildly verified in murine fibrosarcoma, colon carcinoma, and metastatic melanoma models. All the animal studies confirmed the safety of blocking antibodies and proved that CTLA-4 inhibition could lead to potent anti-tumor effects in cancer patients (49).

Regarding survival analysis, high pSTAT3 and high CTLA4 H-scores were significantly associated with shorter OS. In agreement with us**, **Qin *et al.*, Gargalionis *et al.*, and Masuda *et al.* could demonstrate this link at the mRNA level (29, 35, 56). Other solid tumors such as esophageal cancer, breast carcinoma, and nasopharyngeal cancer also showed shorter OS and a worse prognosis when tumor CTLA-4 expression is higher (57-59).

The stage of the tumor, lymphovascular invasion, margins status, tumor stroma ratio, infiltrating tumor border, CEA, CA19.9, initial presentation of IO, pSTAT3, and CTLA4 expression were identified as independent prognostic factors affecting patients' OS in multivariate analysis using the Cox-regression test. These findings are in line with a prior study of CRC cancers (49).

The FDA has approved the use of CTLA-4 inhibitors to treat cancer patients due to their effective outcomes in suppressing a variety of malignancies (60).

According to a recent meta-analysis, CTLA-4 gene expression is associated with a poor prognosis (49). The prognosis of the CRC patients may be improved by focusing on this pro-tumorigenic axis (61). 

The effectiveness and safety of the combination of Nivolumab and Ipilimumab in the treatment of advanced CRC were investigated in a significant phase II clinical research. In total, 120 individuals with metastatic or recurrent CRC, comprising 100 patients with MSI-H and 20 patients with MSS CRC, were enrolled in the trial. According to the findings, Nivolumab alone and Nivolumab plus Ipilimumab had immune response rates of 25.5% (12/47) and 33.3% (9/27) in the MSI-H group, respectively. However, only 5% (1/20) of the MSS group had a PR to combined Nivolumab and Ipilimumab, and no immune response was seen when Nivolumab was applied alone (62, 63).

The phase II CheckMate-142 trial evaluated the role of nivolumab in combination with Ipilimumab for first-line treatment of dMMR/MSI-H mCRC. A 2020 abstract reported results from a longer follow-up showed that the ORR increased to 69%, and the CR rate was 13%. While median PFS and OS had not yet been reached, 24-month rates for these outcome measures were 74% and 79%, respectively (64).

Correlation between both markers showed a positive significant association, which ensures the established role of STAT3 in regulating T cell-mediated cancer progression. Cell-selective targeted therapeutic strategies to inhibit STAT3 activation in T cells are of tremendous interest for future immunotherapies (65)**. **Therefore, combining anti-CTLA-4 and anti-STAT3 treatments improves their functions and induces direct tumor cell killing.

##  Conclusion

STAT3 and CTLA4 positivity may be linked to the development and progression of CRC, and they may provide potential prognostic indicators and therapeutic targets for CRC patients.

## Conflict of Interest

The authors declared no conflict of interest.
